# Secondary Metabolites from the Endoparasitic Nematophagous Fungus *Harposporium anguillulae* YMF 1.01751

**DOI:** 10.3390/microorganisms10081553

**Published:** 2022-07-31

**Authors:** Zebao Dai, Yang Gan, Peiji Zhao, Guohong Li

**Affiliations:** State Key Laboratory for Conservation and Utilization of Bio-Resources in Yunnan, Key Laboratory for Southwest Microbial Diversity of the Ministry of Education, Yunnan University, Kunming 650091, China; yyg0517@163.com (Z.D.); ganyang01@126.com (Y.G.); pjzhao@ynu.edu.cn (P.Z.)

**Keywords:** endoparasitic nematophagous fungi, *Harposporium anguillulae*, scanning electron microscopy, *Meloidogyne incognita*, secondary metabolites

## Abstract

*Harposporium anguillulae*, an endoparasitic nematophagous fungus (ENF), is a model fungus from which the genus *Harposporium* was established. It can infect nematodes via ingested conidia. In this paper, the morphology and nematode–fungus interaction between *Panagrellus redivivus* and *H. anguillulae* were observed by scanning electron microscopy (SEM). The secondary metabolites of *H. anguillulae* were also studied. Seven metabolites were purified and identified from an ethyl acetate extract of broth and a methanol extract of mycelium. These include a new polyketone 5-hydroxy-3-(hydroxymethyl)-6-methyl-2*H*-pyran-2-one (**1**) and six known metabolites (17*R*)-17-methylincisterol (**2**), eburicol (**3**), ergosterol peroxide (**4**), terpendole C (**5**), (3β,5α,9β,22*E*)-3,5-dihydroxy-ergosta-7,22-dien-6-one (**6**), and 5α,6β-epoxy-(22*E*,24*R*)-ergosta-8,22-diene- 3β,7α-diol (**7**). These metabolites were assayed for their activity against plant root-knot nematode, *Meloidogyne incognita*, and the results showed that terpendole C (**5**) had weak nematicidal activity but also that other compounds did not have evident activity at a concentration of 400 μg mL^−^^1^. Compound **1** exhibited an attractive effect towards *P. redivivus*.

## 1. Introduction

The diseases caused by plant parasitic nematodes lead to huge economic losses to crops every year [1]. Traditional chemical pesticides to control nematode diseases have irreversible damaged the ecological environment, so biological control has gradually become a hot topic of research. Nematophagous fungi play an important role as the main source of nematode biological control management [2]. Nematophagous fungi include trapping fungi, endoparasitic nematophagous fungi (ENF), opportunistic fungi, and toxic fungi [3]. Among them, ENF are a type of nematophagous fungi that can parasitize nematodes through special spores.

Fungi of the genus *Harposporium* are a class of endoparasitic nematophagous fungi that can produce conidia to parasitize nematodes, and their unique spore morphology provides more possibilities for them to exhibit nematicidal effects [4]. *Harposporium* was established by Lohde (1874) [5], and the genus is an important group of endoparasitic nematophagous fungi. *Harposporium* species are widely distributed and can be isolated from soil and substrate nematodes [6,7]. Up to now, 31 species of *Harposporium* have been reported all over the world [8]. In addition to producing infection conidia, some species can also produce arthroconidia, chlamydospores, and accessory conidia [9]. In studies of *Harposporium* metabolites, only one paper has reported a new furan, harposporin A, along with a known aureonitol that was found in *Harposporium* sp. YMF 1.01735 [10].

*H. anguillulae* was the first fungus described as a parasite of nematodes. It is also the model fungus from which the genus *Harposporium* was established. It can infect nematodes by producing abundant ingested conidia. The process from ingesting conidia to infecting nematodes is divided into three processes: ingestion, germination, and penetration. *H. anguillulae* can form spores and chlamydospores in water–agar petri dishes. In an investigation of phospholipids on growth and sporulation of *H. anguillulae*, the result showed that the mycelial growth and sporulation of the species was not affected by the addition of phospholipids [11]. *H. anguillulae* can significantly reduce the free living stages of trichostrongylid nematodes, indicating that the species could be a candidate for the development of a biocontrol agent for trichostrongylid nematodes [12]. 

In the present investigation, research on *H. anguillulae* mainly focuses on strain isolation, identification, and pathogenicity to nematodes, in addition to studying secondary metabolites, which have not been reported for *H. anguillulae*. In this study, the strain *H.* *anguillulae* YMF 1.01751 was selected as the research object for its morphology and secondary metabolites, in which seven compounds, including one new polyketone, were identified.

## 2. Materials and Methods

### 2.1. Normal Materials

*H. anguillulae* YMF 1.01751 was isolated from soil in Yunnan Province, China, and is now deposited in the microbial library of the germplasm bank of wild species from Southwest China, Yunnan University, Kunming, China. It was stored in glycerol at –80 °C.

The plant root-knot nematode *Meloidogyne incognita* was obtained from the roots of tomatoes grown in E’shan County in Yunnan Province. The method of obtaining *M. incognita* is referred to in the literature [13].

*Panagrellus redivivus*: An appropriate amount of oat (25 g) and water (60 mL) was added to a 250 mL conical flask for sterilization. After cooling, an appropriate amount of *P. redivivus* seeds were picked and inoculated into oat medium and cultured at 25 °C for one week. The culture and preparation of *P. redivivus* is referred to in the literature [14].

### 2.2. General Experimental Instruments

Optical rotation was measured with a Jasco DIP-370 digital polarimeter (Tokyo, Japan). Ultraviolet (UV) spectra were recorded on a Shimadzu UV-2401PC spectrophotometer (Kyoto, Japan). Nuclear magnetic resonance (NMR) spectra were measured on an Avance III-600 spectrometer (Bruker Biospin, Rheinstetten, Germany). Electrospray ionization mass spectrometry (ESI–MS) spectra were recorded on a Thermo high-resolution Q Exactive Focus mass spectrometer (Thermo, Bremen, Germany). Column chromatography was performed on silica gel G (200–300 mesh, Qingdao Marine Chemical Inc., Qingdao, China) and Sephadex LH-20 (Amersham Biosciences, Piscataway, NJ, USA) columns.

### 2.3. The Morphology and Pathogenicity against Nematodes of H. anguillulae YMF 1.01751 Observed by Scanning Electron Microscopy 

*H. anguillulae* YMF 1.01751 was cultured on improved potato dextrose agar (PDA) (200 g potato, 20 g glucose, 3 g KH_2_PO_4_, 1.5 g MgSO_4_, 5 g yeast extract, 2 g peptone, and 15 g agar) plates for 14 days. *P. redivivus* was cultured in oats for about 7 days, and the oats containing nematodes were placed into three layers of sterilized lens paper, which were then placed into sterile water to allow the nematodes separate from the oats. The nematodes were prepared as a suspension in water corresponding to about 100 nematodes/10 μL.

After growth of *H. anguillulae* YMF 1.01751 on improved PDA plates for 14 days, 20 μL *P. redivivus* suspension was added to the cultured strains for interaction. Samples were taken after 12, 24, 36, 48, 60, and 72 h of nematode–fungus coculture on plates. These samples were cut into small pieces and fixed for 30 min in 4% glutaraldehyde, then dehydrated with 30%, 50%, 70%, 80%, and 90% ethanol for 15 min each, respectively, and then dehydrated twice with 100% ethanol for 15 min each time, then, after that, in ethanol/isoamyl acetate (1:1, *v*/*v*) and in 100% isoamyl acetate liquor for 10 min, respectively. The samples were freeze-dried, gilded, and observed by scanning electron microscopy (SEM) [15].

### 2.4. Extraction and Isolation of Metabolites from H. anguillulae YMF 1.01751

*H. anguillulae* YMF 1.01751 was grown on 50 L optimized liquid medium (200 g potato, 20 g glucose, 3 g KH_2_PO_4_, 1.5 g MgSO_4_, 5 g yeast extract, 2 g peptone) at 25 °C for 21 days. The mycelia and fermentation broth were separated using 5 layers of gauze. The fermentation broth was concentrated to 2 L under vacuum and then transferred to a 5 L separation funnel. The crude extracts (10.3 g) extracted with ethyl acetate were fractionated with a silica gel G (200–300 mesh) column eluted using a petroleum ether–ethyl acetate (100:1-0:100) solvent system followed by trichloromethane–acetone (7:3) and trichloromethan–methanol (7:3) to yield 10 fractions (T1751-1~T1751-10). Dried mycelia were soaked in methanol for 5 days, and the metabolites were repeatedly extracted 3 times. The methanol phase was evaporated in vacuo to provide the mycelium extracts (50 g). The extracts were fractionated with a silica gel G (200–300 mesh) column eluted with a petroleum ether–acetone (100:1-0:100) gradient solvent system followed by ethyl acetate–methanol (10:1-0:1) to yield 16 fractions (B1751-1~B1751-16).

T1751-7 (920 mg) was subjected to a Sephadex LH-20 column eluted with methanol to yield 8 fractions, namely 7-1~7-8. Fraction 7-4 (350 mg) was purified by a silica gel column eluted with ethyl acetate–acetone (20:1-8:1) to produce 6 portions (7-4-1~7-4-6). Compound **1** (5 mg) was obtained from 7-4-1 (12 mg) with a methanol Sephadex LH-20 column. B1751-2 (205 mg) was subjected to a Sephadex LH-20 column eluted with methanol to yield 2-1 (52 mg) and 2-2 (30 mg). Fraction 2-1 was submitted to a silica gel column eluted with ethyl acetate–acetone (150:1) to obtain 2 fractions (2-1-1~2-1-2). These two fractions were each purified by an acetone Sephadex LH-20 column to obtain compounds **2** (6 mg) and **3** (3 mg). B1751-4 (97 mg) was submitted to a silica gel column eluted with ethyl acetate–acetone (120:1-60:1) to produce 3 fractions (4-1~4-3). Fraction 4-3 (9.6 mg) was isolated by a silica gel column eluted with dichloromethane–acetone (50:1-40:1) to yield 2 fractions (4-3-1~4-3-2). Compound **4** (8 mg) was obtained from 4-3-1 (8.5 mg) by a Sephadex LH-20 column eluted with trichloromethane–methanol (1:1, *v/v*).

B1751-8 (161 mg) was subjected to a methanol Sephadex LH-20 column to yield 3 fractions, namely 8-1~8-3. Fraction 8-3 (129 mg) was separated by a silica gel column eluted with ethyl acetate–acetone (50:1-5:1) to produce 4 portions (8-3-1~8-3-4). Fraction 8-3-3 (48 mg) was purified by a Sephadex LH-20 column eluted with methanol to yield 2 fractions (8-3-3-1 and 8-3-3-2). Fraction 8-3-3-1 (40 mg) was isolated by a silica gel column eluted with dichloromethane–acetone (50:1-5:1) and then purified on a methanol Sephadex LH-20 column to provide compound **5** (4 mg). B1751-11 (30 mg) was subjected to a methanol Sephadex LH-20 column to yield 3 fractions, namely 11-1~11-3. Fraction 11-3 was isolated on a silica gel column eluted with petroleum ether–acetone (10:1-3:1) to produce 3 fractions (11-3-1~11-3-3). Compounds **6** (1.7 mg) and **7** (2 mg) were obtained from 11-3-1 and 11-3-3 with a methanol Sephadex LH-20 column respectively.

### 2.5. Nematicidal and Chemotaxis Activity of Metabolites

The tested compounds (**1**, **2**, **3**, and **5**) were dissolved in methanol and diluted with sterile water. Approximately 200 *M. incognita* nematodes J2s were added to each plate, and the final concentration of compounds was 400 µg mL^−^^1^. *M. incognita* was considered dead when no movement occurred after touching it with a needle [16]. The mortality was calculated at different times, and the experiment was repeated 3 times.

The nematode chemotaxis assay for compounds **1**, **2**, **3**, and **5** was conducted using the four-point plate method [17]. The compounds’ concentrations were set to 4, 8, 16, and 32 µg. Approximately 200 worms were added to the center of the petri dish. Compounds and control (methanol) were added. After 2 h at 25 °C, the worms were counted under the microscope, and the chemotaxis index (CI) was calculated.

## 3. Results

### 3.1. Scanning Electron Microscopy Observation of Interactions between H. anguillulae YMF 1.01751 and P. redivivus

**Figure 1:**
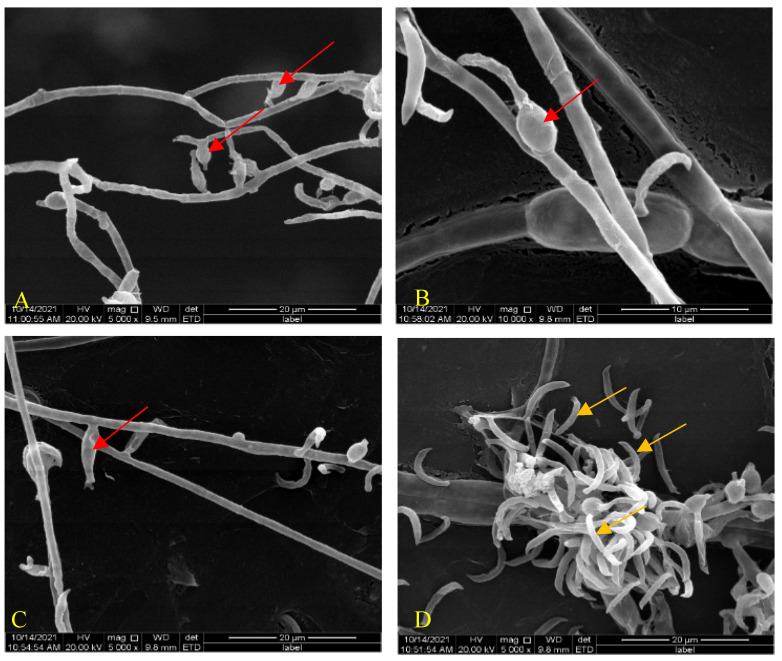
Morphology of *H. anguillulae* YMF 1.01751. (**A**–**C**) Phialides produced by *H. anguillulae* YMF 1.01751 (red arrow: phialides). (**D**) Conidia produced by *H. anguillulae* YMF 1.01751 (orange arrow: conidia).

After growth on the improved PDA medium for two weeks at 28 °C, the hyphae of *H. anguillulae* YMF 1.01751 had overspread 6 cm on the plate. The mycelia of *H. anguillulae* YMF 1.01751 were septate and colorless. SEM results show that the phialides (sporogenous cell) of *H. anguillulae* YMF 1.01751 were globose or flask-shaped, and there was a conidial peduncle on the tip of phialides. Conidia were colorless, curved, bow-shaped, and had hooked ends (Figure 1A–D).

*H. anguillulae* produces ingested conidia to infect nematodes. While studying the infection of *P. redivivus* by *H. anguillulae* YMF 1.01751, approximately 200 worms were added to each plate. In the process of infesting nematodes, the conidia were swallowed by nematodes, after which the spores underwent germination to produce hyphae, which penetrated the body wall of the nematodes and stuck there for further growth to produce new conidia again. If nematodes were present, a new round of the infestation process could be carried out.

**Figure 2:**
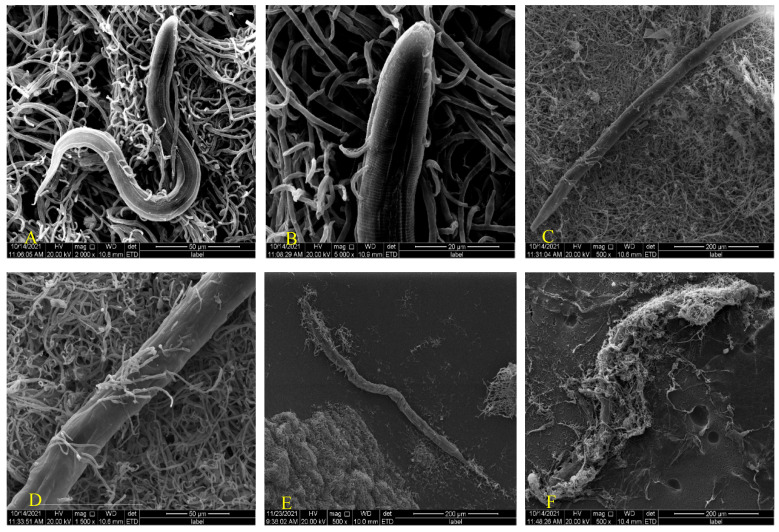
Scanning electron microscopy observation of the interaction between *H. anguillulae* YMF 1.01751 and *P. redivivus*. (**A**) The coculture of *H. anguillulae* YMF 1.01751 and *P. redivivus*. (**B**) The hyphae begin to grow through nematode body. (**C**,**D**) A lot of hyphae continue to grow through the body wall. (**E**,**F**) The nematode was finally surrounded by hyphae.

Endoparasitic nematophagous fungi can infect nematodes by producing adhesive conidia or ingested conidia. Adhesive conidia were specifically identified and adhered to the body wall, head, and genital pore of the nematode, then grew and reproduced using nematodes. Previous studies have shown that treating the nematode epidermis with pronease E can cause the conidia to fail to adsorb, which leads to the inference that nematode surface proteins play a key role in the inter action process of fungi and nematodes. Similarly, trypsin treatment for conidia eventually resulted in a reduction in the ability of the conidia to adsorb onto nematodes. It has been speculated that the interaction process of nematode and adhesive conidia is a protein-with-protein process of interaction [18]. The process by which ingested conidia infect nematodes is the same as the interaction between *H. anguillulae* YMF 1.01751 and nematodes, which is divided into swallowing conidia, parasitism, germination, and penetration. This interaction begins when the nematode ingests conidia [9]. In addition to being swallowed, some spores will attach to the surface of the nematode (Figure 2A). The conidia began to germinate and the hyphae grew through nematode body about 48 h after being swallowed (Figure 2B), and some nematode had already begun to die. Subsequently, a lot of hyphae would continue to grow through the body wall (Figure 2C,D) until the nematode mass was finally surrounded (Figure 2E,F), and new conidia were then produced. A new round of infestation was performed in the case of nematodes.

### 3.2. Structure Identification

Seven compounds (**1**–**7**) were isolated from an ethyl acetate extract of broth and a methanol extract of the mycelium of *H. anguillulae* YMF 1.01751. Their structures were identified by nulear magnetic resonance (NMR) and mass spectrometry (MS) data.

**Figure 3:**
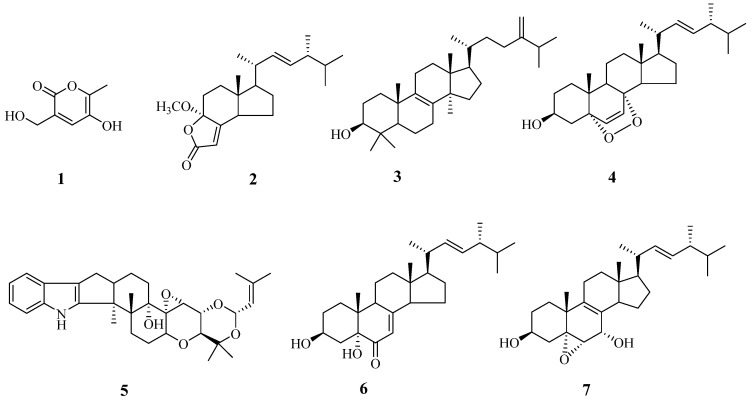
The compounds isolated from *H. anguillulae* YMF 1751.

Compound **1** was obtained as colorless solid. The molecular formula of compound **1** is C_7_H_8_O_4_ (157.0494 [M + H]^+^, Calcd. 157.0495), which was calculated with positive ion mode high-resolution mass spectrometry. It contains four unsaturation degrees. According to ^13^C-nulear magnetic resonance and distortionless enhancement by polarization transfer (DEPT) (Table 1) data, compound **1** contains one methyl, one oxygen-substituted methylene, one methine, and four quaternary carbons. Based on two-dimensional nuclear magnetic resonance (2D-NMR) data, H-6 (δ_H_ 2.36) is correlated with C-5 (δc 141.9) and C-4 (δc 148.7), H-3 (δ_H_ 7.59) is related with C-4 (δc 148.7), C-1 (δc 168.2) and C-2 (δc 146.3), and H-7 (δ_H_ 3.79) is correlated with C-2 (δc 146.3). Thus, compound **1** was identified as 5-hydroxy-3-(hydroxymethyl)-6-methyl-2*H*-pyran-2-one, and its structure is shown in Figure **3**.

Compounds **2**–**7** were determined to be (17*R*)-17-methylincisterol (**2**) [19], eburicol (**3**) [20], ergosterol peroxide (**4**) [21], terpendole C (**5**) [22], (3β,5α,9β,22*E*)-3,5-dihydroxy-ergosta-7,22-dien-6-one (**6**) [23] and 5α,6β-epoxy-(22*E*,24*R*)-ergosta-8,22-diene-3β,7α-diol (**7**) [24]. Their structures are shown in Figure 3.

### 3.3. The Nematicidal and Chemotaxis Activity of Compounds 

Compounds **1**, **2**, **3**, and **5** were tested for their nematicial activity against root-knot nematode *M. incognita*. The results showed that terpendole C (**5**) caused 37.4% mortality of *M. incognita* at 400 μg mL^−1^ during 48 h. Other compounds have no evident nematicidal activity at the same concentration. Terpendole C (**5**) is an indole diterpene. This type of compound has been reported to have nematicidal activity. A similar compound, gymnoascole acetate, was obtained from fungus *Gymnoascus reessii* za-30 and caused 100% paralysis of *M. incognita* at 36 μg mL^−1^ for 24 h [25].

**Figure 4:**
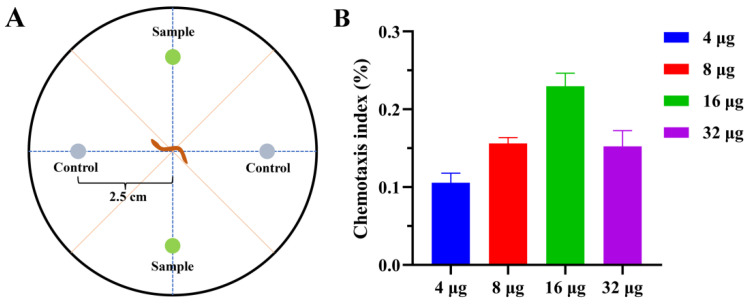
Effect of compounds on the chemotaxis of *P. redivivus*. (**A**) Schematic representation of the quadrant bioassay used to measure the chemotaxis of compounds. (**B**) Chemotaxis activity of **1** at different concentrations.

In the chemotaxis assay, the new polyketone **1** exhibited an attractive effect towards *P. redivivus*. With the concentration increased (4, 8, 16 µg), the attraction activity of compound **1** was enhanced. At 16 µg, it showed a chemotaxis index of 0.21 after 2 h. The attractive effect of compound **1** decreased with increasing concentration (32 µg) after 2 h (Figure 4). The other tested metabolites (**1**, **2,** and **3**,) displayed no pronounced activity.

## 4. Discussion

In this study, the morphology and pathogenicity of *H. anguillulae* YMF 1.01751 was observed by scanning electron microscopy. It produces abundant crescent-shaped conidia. This kind of long and curved spore is characteristic of *Harposporium*. Most *Herposporiums* infect nematodes by ingested spores that germinate inside the nematode and produce septate and branching hyphae that absorb the nutritious of the nematode. At last, the hyphae break out through the nematode cuticle [26].

The fungal metabolites were studied. Seven metabolites, include a new polyketide compound 5-hydroxy-3-(hydroxymethyl)-6-methyl-2*H*-pyran-2-one (**1**), were isolated from a fermented extract of *H. anguillulae* YMF 1.01751. Polyketide **1** exhibited an attractive activity towards *P. redivivus*. This is the first report about the genus *Harposporium* secreting small molecular metabolites to attract nematodes. Some biocontrol potential microorganisms can attract nematodes and further kill them. *Bacillus nematocida* B16 produces 2-heptanone to attract nematodes, subsequently killing them through protease secretion [27]. 

Other structures of the known metabolites are triterpene, indole diterpene, and steroid. (17*R*)-17-Methylincisterol (**2**) was identified from *Dictyonella incisa* for the first time as a new natural product. It is believed that the biosynthesis pathway of this compound might begin with the unique oxidation process of cholestatrien-3β-ols in vivo [15]. In addition, the compound can also be obtained from the fungus *Aspergillus terreus* TZS-201607 and has certain cytotoxic activity against A549, MCF-7, and THP-1 with IC_50_ values of 48.9, 16.8, and 23.6 μM, respectively [28]. Compounds **4**, **6**, and **7** are sterols. Steroids are a kind of important natural organic compounds that are widespread in biological tissues. With cyclopentane polyhydrophenanthrene as the mother nucleus, steroids can be divided into many types according to their substituents, double-bond positions or stereoconfigurations. Steroids have a variety of biological activities because of their structural specificity, such as antitumor, anti-inflammatory, antibacterial, antiviral, and enzyme-inhibitory activities [29]. Compound **6** showed no activity in an experiment evaluating neutrophil elastase (HNE) activity [30]. Compound **7** exists in a variety of fungi and has moderate cytotoxic activity on tumor cells such as A549 (non-small cell lung adenocarcinoma), SK-OV-3 (ovarian cancer), SK-MEL-2 (skin melanoma), XF498 (central nervous system), and HCT15 (colon) cultured in vitro. In addition, compound **7** can inhibit the growth of the hypocotyl of lettuce and promote the growth of lettuce root at the same concentration [31].

Terpendole C (**5**) is an indole diterpene, and many indole diterpenoids have been reported to be metabolites of fungi. Terpendole C (**5**) showed acyl-CoA: cholesterol acyltransferase (ACAT)-inhibitory activity, with an IC_50_ value of 2.1 μM, in an vitro assay [32]. Terpendole C (**5**) can specifically inhibit the synthesis of cholesterol esters in peritoneal macrophages by inhibiting cholesterol acyltransferase activity [33]. In addition to enzyme-inhibitory activity, the compound also has cytotoxic activity and antibacterial activity. It has a cytotoxic effect on NCI-H187 and Vero cells, with IC_50_ values of 35.69 μM and 23.56 μM, respectively, and it has anti-*Mycobacterium tuberculosis* H37Ra activity, with an MIC value of 25.00 μg mL^−1^ [34]. According to reports, some indole diterpenes have nematicidal activity. For example, gymnoascole acetate, from *G. reessii* za-30, has activity against *M. incognita,* with an EC_50_ value of 47.5 μg mL^−1^ for 24 h [25]. This type of metabolite may be a potential biocontrol resource for nematodes. In this study, compound **5** showed weak activity against nematodes. In the isolation process of metabolites from *H. anguillulae* YMF 1.01751, some other indole diterpenes had not been identified due to their presence in minor amounts. These compounds that have not been isolated may be potential active substances. Therefore, the metabolites of *H. anguillulae* deserve further study.

*H. anguillulae* is a typical endoparasitic fungus of nematodes. It infects nematodes by producing ingested conidia. This is the first report on the metabolites of the species. The results state that terpendole C (**5**) has weak nematicidal activity against root-knot nematode *M. incognita*, and polyketide **1** exhibites an attractive effect towards the nematode. This result indicated that some secondary metabolites were involved in the pathogenicity process of *H. anguillulae* infecting nematodes.

## Figures and Tables

**Table 1:** The nuclear magnetic resonance data of compound **1** in trichloromethane-d (600 MHz).

Position	^1^H	^13^C	HMBC
1	-	168.2, s	-
2	-	146.3, s	-
3	7.59 (1H, s)	138.3, d	148.7, 168.2, 146.3
4	-	148.7, s	-
5	-	141.9, s	-
6	2.36 (3H, s)	14.6, q	141.9, 148.7
7	3.79 (2H, s)	57.0, t	146.3

## Data Availability

Not applicable.
